# Description of Gas Transport in Polymers: Integrated Thermodynamic and Transport Modeling of Refrigerant Gases in Polymeric Membranes

**DOI:** 10.3390/polym17162169

**Published:** 2025-08-08

**Authors:** Matteo Minelli, Marco Giacinti Baschetti, Virginia Signorini

**Affiliations:** Department of Civil, Chemical, Environmental and Materials Engineering, Alma Mater Studiorum, University of Bologna, via Terracini 28, 40131 Bologna, Italy; matteo.minelli@unibo.it (M.M.); marco.giacinti@unibo.it (M.G.B.)

**Keywords:** refrigerant gases, polymeric membranes, EoS modeling, NELF

## Abstract

Hydrofluorocarbons (HFC) are today widely used as refrigerants, solvents, or aerosols for fire protection. Due to their non-negligible environmental impact, there exists an increasing interest towards their effective separation and recovery, which still remains a major challenge. This work presents a comprehensive thermodynamic and transport modeling approach able to describe HFC sorption and transport in different amorphous polymers, including glassy, rubbery, and copolymers, as well as in supported Ionic Liquid membranes (SILMs). In particular, the literature solubility data for refrigerants such as R-32, R-125, R-134a, and R-152a is analyzed by means of the Sanchez–Lacombe Equation of State (SL-EoS), and its non-equilibrium extension (NELF), to predict gas uptake in complex polymeric materials. The Standard Transport Model (STM) is then employed to describe permeability behaviors, incorporating concentration-dependent diffusion using a mobility coefficient and thermodynamic factor. Results demonstrate that fluorinated gases exhibit strong affinity to fluorinated and high free-volume polymers, and that solubility is primarily governed by gas condensability, molecular size, and polymer structure. The combined EoS–STM approach accurately predicts both solubility and permeability across different pressures in all polymers, including SILM. The thorough study of HFC transport in polymer membranes provided both systematic insights and predictive capabilities to guide the design of next-generation materials for refrigerant recovery and low-GWP separation processes.

## 1. Introduction

Refrigerant gases, which belong to the class of fluorinated substances (F-gases), play a crucial role in various industrial and domestic applications- such as air conditioning, cooling system, heat pumps, aerosol and foam blowing agents [1]—thanks to their thermodynamic properties and efficient heat transfer capabilities. However, the widespread use of these synthetic compounds has raised environmental concerns due to their high global warming potential (GWP) and ozone depletion ability. Starting from the Montreal Protocol in 1987 [2], later highlighted by European Union legislation in 2014 [3], halogenated hydrocarbons have been banned due to their detrimental effect on the ozone layer that shields the planet against ultraviolet radiations [4].

These two major environmental concerns have serious implications in the future development of refrigeration-based industries, since new legislation has focused not only on reducing emissions of greenhouse gases, such as hydrofluorocarbons (HFCs) [5], but also on promoting the recovery, reuse and recycling of refrigerants. In this concern, novel separation technologies are of particular interest for purifying HFC refrigerants, with the aim of facilitating the recycling and reuse of low-GWP HFCs, enabling the use of environmentally friendly refrigerant blends [6] that are compatible with existing systems.

In recent decades, membrane technology has garnered increasing interest due to its compactness, operational efficiency, scalability, environmental benefits, and lower energy demand compared to traditional separation techniques. Such attributes have led to its adoption in various applications, including natural gas sweetening [7,8,9], air separation, hydrogen and helium recovery [10,11,12,13,14,15], and CO_2_ capture from flue gas [16,17,18,19] or syngas adjustment [20,21]. Furthermore, research has investigated the potential of membrane technology for the separation of hydrofluorocarbons (HFCs), particularly R-32, R-125, R-152a, and R-134a.

Current studies have explored the use of perfluorinated glassy copolymers, such as perfluoro(butenyl vinyl ether) (PBVE) and perfluoro(2,2-dimethyl-1,3-dioxole) (PDD) [22,23,24], as well as copolymers made of PDD and Vinyl Acetate (VA) [25] and poly(ether-block-amide) (PEBAX) [26,27] for gas separation membranes, as potential candidates for separating refrigerant mixtures, considering R-32 and R-125 as reference gases.

Harders et al. [23] investigated the separation of R-32 from R-410A comparing the performances of rubbery PDMS and a glassy perfluorinated copolymer (CyclAFlor™) membrane. The results demonstrated that while PDMS exhibited significantly higher gas permeability, the 5%PBVE-co-95%PDD copolymer provided superior selectivity, with a mixed-gas separation factor of 13.9 for R-32 over R-125. Moreover, in a later work the same authors [24] characterized in detail PBVE-PDD copolymers with different mass ratios, revealing that their ability to adjust FFV of the copolymer by conveniently tuning the amount of PBVE or PDD, provides interesting gas separation performances of refrigerant mixtures. PBVE, commercially known as Cytop [28], is an amorphous perfluoropolymer with high permeability with respect to common light gases, good chemical stability, and resistance to plasticization [29]. PDD, on the other hand, is a monomer that contains bulky CF_3_ groups into the polymer backbone that causes a significantly increase in its free volume [30]. The combination of these two material properties is designed to limit chain mobility and restrict the diffusion of larger gas molecules, thus improving selectivity of different pair of gases, such as He/CH_4_, He/H_2_, N_2_/CH_4_ and H_2_/CH_4_ [31], making them suitable also for the separations of larger molecules [29]. Similarly, PEBAX membranes have demonstrated significant potential for HFC and HFO (hydrofluoro olefin) separation due to their dual-phase structure comprising a flexible polyether phase and a rigid polyamide segment. Such morphology provides an optimal balance between permeability and selectivity, with reported permeability values reaching up to 200 Barrer for R-32, and selectivity values as high as 10 for R-32/R-1234 yf mixture [26,27].

In a different study, Gutierrez-Hernández et al. [32] demonstrated that Polymer of Intrinsic Microporosity (PIM-1) membranes exhibit exceptional permeability and selectivity for fluorinated refrigerant gases, achieving CO_2_-like permeation rates of up to 4100 Barrer for difluoromethane (R-32) and R-32/R-125 ideal selectivity up to 28. These findings highlight the potential of PIM-1 for recovering high-value refrigerants from mixed waste streams, particularly in separating R-32 from its close-boiling counterparts.

While research on membrane permeability has advanced, the sorption behavior of refrigerant gases remains relatively underexplored, despite the importance of independently understanding solubility and diffusivity for process optimization. Gas transport in polymeric membranes is indeed governed by solution-diffusion mechanism, driven by specific interactions at the molecular level. Although Flory–Huggins (for rubbers) and dual-mode sorption, DMS (for glasses) have been widely employed to describe gas solubility at low to medium pressures, their application at high pressure and integration with transport properties remains limited. Due to their empirical nature, these models offer limited predictive capabilities, particularly under the high-pressure and low-temperature conditions typical of refrigeration cycles [33]. In contrast, thermodynamic models such as Sanchez–Lacombe lattice fluid [34,35,36], Non-Equilibrium Lattice Fluid (NELF) [37,38,39,40], and PC-SAFT have demonstrated applicability across a wide range of polymer–gas systems and blends [41,42,43,44,45]. Notably, their development has advanced to the point where they are capable not only of describing experimental sorption data, but also of predicting the behavior of both polymers and penetrants over broad temperature and pressure ranges.

Understanding the sorption and transport behavior of hydrofluorocarbons (HFCs) in polymeric materials is essential for optimizing membrane-based separation processes. Moreover, it is of interest to extend such analysis also to novel systems, such as Ionic Liquids (ILs) and supported Ionic Liquid membranes (SILMs) [46,47,48,49,50] which have been proposed as promising alternatives to conventional membranes due to their selective absorption capacity, low vapor pressure, and high chemical and thermal stability [26,51].

A comprehensive and predictive description of the sorption and permeation behavior of HFCs in polymers and SILMs is indeed essential not only for interpreting experimental trends, but also for supporting the design of more efficient and selective membrane-based separation units.

In this work, we present a comprehensive and extended modeling framework to describe the transport behavior of fluorinated gases in both rubbery and glassy polymers. This approach couples thermodynamic and transport analyses, where Equation of State (EoS) models [33,38] are employed to describe penetrant solubility in polymers. These solubility predictions are then integrated into the Standard Transport Model (STM) [52] to investigate the diffusive behavior of the examined penetrant gases and ultimately enabling the prediction of permeability as a function of upstream pressure. The novelty of this work lies in the systematic integration of well-established EoS-based thermodynamic models (such as Sanchez–Lacombe, NELF) with transport modeling to characterize the sorption and permeation of hydrofluorocarbons (HFCs) across a wide range of conditions. To the best of our knowledge, this is the first study that applies such framework specifically to HFCs, to verify the ability of this approach to describe the experimental behavior of both solubility and permeability properties in a wider range of conditions without the need for additional fitting parameters [41,45,53].

Furthermore, this modeling strategy is extended to emerging membrane systems incorporating Ionic Liquids, for which EoS frameworks offer a promising path to describing both sorption and permeation behavior. The proposed methodology aims not only to elucidate the fundamental mechanisms that govern gas–polymer interactions, but also to serve as a practical predictive tool to support the design of advanced membranes for refrigerant recovery and sustainable separation technologies.

## 2. Theoretical Background

The mass transport of gases, vapors or liquids in polymer membranes can be modeled by an integrated approach that makes use of thermodynamic and diffusive properties. This approach is often implemented through the solution-diffusion model [54], which by coupling Fick’s second law with an appropriate equilibrium condition, relates the permeability of a polymer to the kinetics and thermodynamic properties of the polymer penetrant system. In the case of gases and vapors, this approach is typically simplified by using constant diffusion *D* and solubility coefficient *S* to obtain the well-known relationship:

(1) P=D·S

However, this approach can be made more general and rigorous by replacing the assumption of constant diffusion and solubility coefficients with a more robust formulation that accounts for the variation in these parameters as a function of operating conditions, namely, pressure, temperature, and penetrant concentration in the polymer.

More in detail, the penetrant solubility in polymers can be conveniently described by means of Equation of State (EoS) models, by the resolution of the phase equilibrium problem, using appropriate expressions for the penetrant chemical potential either in the fluid phase or within the polymer. For equilibrium systems, polymer melts or rubbers, the Lattice Fluid (LF) EoS [55,56,57] provides a reliable representation of the gas-polymer mixture in which each molecule is considered as a flexible chain composed of *mers* immersed in a lattice of cubic cells [58,59]. In particular, the model derived by Sanchez and Lacombe [35,36] provides the following expression for the system specific volume (or density):

(2) $$\frac{p}{p^{*}}=\frac{{RT}^{*}}{{MP}^{*}}\rho -\left\{{\left(\frac{\rho }{\rho^{*}}\right)}^{2}+\frac{T}{T^{*}}\left[ln\left(1-\frac{\rho }{\rho^{*}}\right)+\frac{\rho }{\rho^{*}}\right]\right\}$$

where *R* is the ideal gas constant, *M* is the average molar mass of the system and $T^{*},\;p^{*}$ and $\rho^{*}$ are the characteristic parameters of the model. In the lattice fluid representation, indeed, each substance is univocally characterized by three macroscopic parameters $T^{*},\;p^{*}$ and $\rho^{*}$: the characteristic temperature, pressure and closed-packed density, respectively. More in detail, p* can be interpreted as a measure of the strength of intermolecular interactions, while the characteristic temperature T* is the ratio of the interaction energy and Boltzman constant, and ρ* is the ratio of molar mass and closed-pack volume [34,58].

These three parameters, for pure substances, are usually estimated experimentally by the best fit of vapor-liquid equilibrium (VLE) for low molecular weight gas components [59,60], and from standard pressure-volume-temperature (pVT) measurements for polymers [61]. The pure component data can then be extended to mixtures by using appropriate mixing rules suggested by the model itself [34,36,62] with the addition of a single binary parameter as it will be better discussed below.

Based on these parameters, the model also provides the gas solubility in the polymer from the resolution of the phase equilibrium problem, considering the equality of chemical potentials of the penetrant *i* in the gas ($\mu_{i}^{gas}$) and that in the solid polymer phase ($\mu_{i}^{pol}$) [45]. The mathematical expression of the chemical potential of a component in a mixture can be derived from LF EoS [45,63], and it is reported in the following Equation (3), in which subscript *i* stands for the penetrant, while the polymer is identified by the subscript *j* [38]:

(3) $$\frac{\mu_{i}}{RT}=\ln\phi_{i}+\left(1-\frac{r_{i}v_{i}^{*}}{r_{j}v_{j}^{*}}\right)\phi_{j}+r_{i}\frac{v_{i}^{*}}{RT}\tilde{\rho }\phi_{j}^{2}{\Delta p}_{ij}^{*}\;\;\;\;+r_{i}(\frac{T_{i}^{*}}{P_{i}^{*}}\frac{P}{\tilde{\rho }T})+\tilde{\rho }\frac{T_{i}^{*}}{T}+\frac{\ln\tilde{\rho }}{r_{i}}-\left(1-\frac{1}{\tilde{\rho }}\right)ln(1-\tilde{\rho })$$

In this equation, $\tilde{\rho }$ is the reduced density ($\tilde{\rho }=\rho /\rho^{*})$, while r is the number of lattice cells occupied (component *i* or *j*, respectively), *ϕ* is a measure of the volume fraction of component *i* or *j* and ${\Delta p}_{ij}^{*}$ accounts for polymer-penetrant specific interaction through the binary interaction parameter $k_{ij}$ that accounts for the energetic interactions between component *i* and *j* in the mixture:

(4) $${\Delta p}_{ij}^{*}=p_{i}^{*}+p_{j}^{*}-2\left(1-k_{ij}\right)\sqrt{p_{i}^{*}p_{j}^{*}}$$

The equation needs to be coupled with that for the chemical potential of the pure penetrant (in the fluid phase) to calculate the equilibrium composition, and to Equation (2) for the representation of the properties of the two substances.

For glassy systems, the SL EoS cannot be readily applied because their nonequilbrium nature prevents the use of correlation between classical state variables (Equation (2)). In this case, indeed, pseudo-equilibrium conditions, characterized by a lower density and a larger free volume than true thermodynamic equilibrium are reached. It has been shown, however, that glassy polymers are well described by the nonequilibrium thermodynamics model proposed by Sarti and Doghieri in 1996 [37], which in the Lattice Fluid framework can be referred to NELF [38]. The main result of the NELF is that the equilibrium expression of the Helmholtz free energy can be extended to represent the nonequilibrium state of the glass by accounting for the actual density as an internal state variable due to the nonequilibrium swelling induced by the penetrant. The non-equilibrium density ρ*^NE^* of the polymer is thus used to measure the departure from equilibrium conditions.

Similar to the case of rubbery materials, calculations on gas solubility in non-equilibrium glassy polymers are performed by imposing equality of the chemical, as the phase equilibrium condition between the gas and the polymer phase holds true, but considers density ($\rho_{pol}^{NE}$) as an additional state variable [37,64].

(5) $$\mu_{i}^{NE\left(pol\right)}\left(T,p,\omega_{i},\rho_{pol}^{NE}\right)=\mu_{i}^{EQ\left(gas\right)}\left(T,p,\omega_{i}\right)$$

Nevertheless, it is well known that polymer density changes upon sorption of condensable gases and this variation must be considered in the resolution of NELF equilibria. In the case of unknown experimental measurements, the polymer swelling can be estimated through a linear relationship between the polymer volume and gas pressure [41,65], as often observed experimentally:

(6) $$1/\rho_{pol}^{NE}=\frac{1}{\rho_{pol}^{0}}\left(1+k_{sw}p\right)$$

where p is the partial pressure of the penetrant, $\rho_{pol}^{0}$ is the unpenetrated polymer density and $k_{sw,i}$ is the swelling coefficient of the species in the polymer, calculated from the analysis of the solubility isotherms [38,66,67].

While model analyses have mainly been applied to describe the solubility of pure penetrants and gas mixtures in homopolymers, many applications involve the use of polymer blends and copolymers. Even in these cases, however, thanks to the solid theoretical foundation of this modeling approach, extending the model is relatively straightforward and reliable [66,68]. All model parameters are indeed physically meaningful, allowing the solubility isotherms in miscible copolymers to be derived from the knowledge of pure component pVT values using appropriate mixing rules, visible in Table S1 of the Supplementary Materials, that have been proven to work effectively not only for the polymer-solvent systems, but also for blends of two polymeric phases [42].

The use of LF/NELF EoS allows the description and the prediction of the solubility of refrigerant gases in glassy and rubbery polymer and copolymers, and to the determination of the binary interaction parameter *k_ij_* and swelling coefficient *k_sw_*.

In order to further improve the consistency and the predictive behavior of SL-LF EoS, this approach was extended also to a more complex system, like polymeric matrices containing a certain amount of Ionic Liquids. To assess the accuracy and practical utility of the proposed method, various solubility isotherms of different refrigerant gases (the results obtained are reported in Supplementary Materials File), namely R-32, R-125 and R-134a, have been modeled in [C_2_mim][BF_4_] and [C_2_mim][SCN], considering the phase equilibrium between the two fluids phases, while ignoring any chemical interaction between ILs and fluorinated refrigerant gases [69,70,71,72]. Once the characteristic parameters for ILs are obtained, the polymer and IL blends are combined into a single multicomponent phase using standard mixing rules (Table S1), in order to determine the EoS coefficients for polymer + ILs systems. VLE data has been retrieved from literature for each IL considered in order to calculate the characteristic parameters required by SL-Lattice Fluid EoS (Figures S2 and S3 in the Supplementary Materials).

Furthermore, the Standard Transport Model (STM) has been proposed to describe the gas diffusion and transport in polymers over a broad range of temperature and pressure [52,73,74]. It considers that the mass flux of a penetrant gas through a polymeric layer is obtained by considering the penetrant chemical potential gradient, the process driving force and the mobility coefficient, L, often also recalled as thermodynamically corrected diffusivity. Such quantity accounts for the resistance encountered by molecular motion inside the solid layer, and it is related to the properties of the pure polymer, those of the pure penetrant, as well as the concentration of the penetrant dissolved in the matrix. The latter dependence may be conveniently described by a simple exponential dependence of *L* on gas concentration mass fraction, ω, in the polymer [52,74]:

(7) $$L=L_{\infty }e^{\beta \omega }$$

where $L_{\infty }$ is the infinite dilution mobility coefficient and β is the plasticization factor, which represents the increase in *L* inside the polymer upon plasticization and swelling. As said above, the mass transport in polymers is usually described by Fick’s law [74,75], and coupling it with the definition of mobility coefficient, one can define the diffusion coefficient as follows:

(8) $$D=L\frac{\partial \mu_{i}/RT}{\partial ln\omega_{i}}=L\alpha$$

in which α is the thermodynamic factor, defined as the ratio of the chemical potential gradient and the gas solubility (expressed as mass fraction) in the membrane: $\alpha =\frac{\partial \mu_{i}/RT}{\partial ln\omega_{i}}$, which can be obtained directly from experimental solubility isotherms or from thermodynamic solubility models [76,77,78].

Finally, the gas permeability (see references [73,79,80,81,82]) can be calculated by coupling together the LF EoS model results with the Fick’s Law, as follows [83]:

(9) $$P_{i}=\frac{1}{\left(p_{i}^{u}-p_{i}^{d}\right)}\int_{p_{i}^{d}}^{p_{i}^{u}}L_{\infty }e^{\beta \omega_{i}}S_{i}z_{i}dp$$

where $p_{i}^{u}$ and $p_{i}^{d}$ are the upstream and downstream pressure of the gas *i* at the two sides of membrane layer, respectively; *S_i_* is the solubility coefficient ($\omega_{i}/p$) of component *i* in the polymer phase, achieved from the phase-equilibrium resolution as a function of pressure (Sanchez Lacombe or NELF model), while *z_i_* is the penetrant compressibility factor of the pure penetrant *i* (calculated using the Peng Robison EoS [84]). Equation (9) therefore represents the equivalent of Equation (1) when robust and theoretically sound models are used to replace the assumption of constant diffusivity and solubility coefficients.

## 3. Results

### 3.1. Model Parameters

The present work aims to extend the range of applicability of the modeling framework in terms of different new materials, gas penetrants, and blends.

The determination of the characteristic parameters (T*, p* and ρ*) of the Lattice Fluid equation is the first step of the modeling activity, the different procedures considered are here briefly recalled, while more detailed data can be found in the SI. While the characteristic parameters for the refrigerant gases are retrieved by the best-fitting (Figure S1 in Supplementary Materials) of Liquid-Vapor Equilibrium (VLE) data [60], the ones for commercial polymers already studied, such as PIM-1 [85], PMMA [53], PS [64], and PDMS [86] are retrieved from the corresponding technical literature. Conversely, the parameters for PVBE-PDD copolymers are estimated by weighing the pure parameters of PBVE [28] and PDD with the respective mass fractions of the two components, according to EoS mixing rules [66,68]. Due to the lack of pVT data, the PDD characteristics parameters are obtained by regressing literature data of two different Poly(2,2-bistrifluoromethyl-4,5-difluoro-1,3-dioxole-*co*-tetrafluoroethylene) copolymers, namely Teflon AF1600 and Teflon AF2400 (made of PDD-*co*-TFE) [30,87], according to thermodynamic mixing rules for copolymer in the EoS framework (Table S1). Similar procedure is performed for PDD-VA and PEBAX 1657 copolymer, where the parameter sets for Poly (ethylene oxide) (PEO) and VA are recovered from literature [88] while polyamide (PA) characteristic triplet is obtained by available pVT data for the pure polymer in the molten state [61]. All characteristic parameters used in this work are summarized in Table 1.

The thermodynamic model has been applied to a variety of different cases retrieved in the literature for refrigerant gas uptake, ranging from glassy polymer to copolymers, SILMs and Ionic Liquids, and across a broad range of thermodynamic conditions —including dense gas and liquid-like states, as reported in the Supplementary Materials (Figures S4–S7)—thus providing deeper insights into polymer–refrigerant interactions under realistic design scenarios. To the best of our knowledge, the application of the Lattice Fluid (LF) theory to describe the sorption behavior of hydrofluorocarbon refrigerants in these classes of polymers has not been reported in the recent literature.

### 3.2. Solubility of R-32

**Figure 1:**
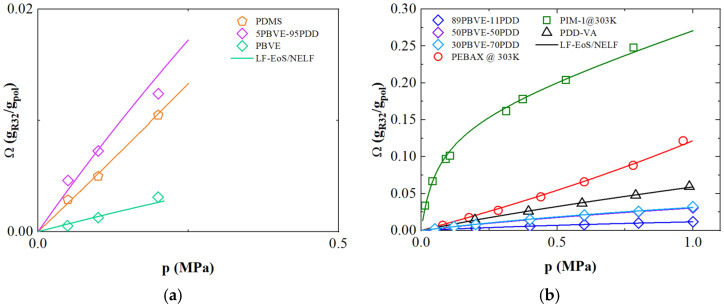
R-32 solubility isotherms (expressed in mass ratio) as a function of pressure for different polymers: (**a**) low pressure; (**b**) high pressure. The symbols represent the experimental data retrieved from literature [23,24,25], while continuous lines are obtained using LF/NELF model.

Figure 1 reports the solubility isotherms (expressed as the mass ratio of the penetrant gas over the mass of the polymer Ω) of R-32 (difluoromethane, CF_2_H_2_) as a function of pressure for different polymers, at 303 K for PIM-1 and PEBAX 1657, and 308 K for all the other materials. The experimental data retrieved from literature [23,24,25] were analyzed by means of the Lattice Fluid EoS for rubbers (PDMS and PEBAX 1657), while the NELF approach was used to describe the other (glassy) polymers. It can be observed that the estimation given by the thermodynamic model (lines in Figure 1) described well the gas uptake in amorphous polymers, both for homopolymers and copolymer materials, with the use of one (for rubber) or two for glassy polymers, adjustable parameters for each curve. The values of the different parameters are reported in Table 2.

Among the different polymers, PBVE presents the lower solubility compared to the other materials, while PIM-1 shows the largest gas uptake. It is interesting to note that the R-32 solubility in copolymers made of PBVE-PDD is enhanced by increasing PDD content in the matrix. The binary interaction parameter *k_ij_* presents a decreasing monotonous trend, with a reduction from 0.10 to 0.049 passing from pure PBVE to 5% PBVE-95%PDD, indicating more positive interaction of the refrigerant with the PDD units. On the other hand, swelling coefficient in PBVE-PDD experienced an increase from PBVE (*k_sw_* = 0.01 MPa^−1^) to 5% PBVE-95%PDD (*k_sw_* = 0.05 MPa^−1^), meaning that the greater FFV given by PDD concentration in the copolymers holds for higher sorption and dilation.

Although the PDD-VA copolymer shows a higher gas uptake (up to 0.06 g_gas_/g_pol_ at 1 MPa) compared to other glassy polymers, it exhibits a lower binary interaction parameter (*k_ij_* = 0.057) than its PBVE-PDD counterpart with similar PDD content. This suggests greater compatibility with R-32. The higher swelling coefficient (0.058 MPa^−1^) observed in the PDD-VA system can be attributed to enhanced matrix polarity, likely due to the presence of vinylamine moieties.

As already mentioned, the largest R-32 sorption capacity is observed in Polymer of Intrinsic Microporosity (PIM-1), as reported by Gutierrez et al. [32]. Thanks to its rigid and twisted structure, this polymer is characterized by a free volume significantly larger than the other glassy polymers inspected, which leads the R-32 concentration to 0.25 g_gas_/g_Ppol_ at 0.9 MPa. PIM-1 was modeled using the NELF approach, identifying a *k_ij_* value equal to 0.06 and a *k_sw_* of 0.09 MPa^−1^.

Regarding the rubbery materials, PDMS shows a lower solubility with respect to PEBAX 1657, although comparable to that of 5%PBVE-95%PDD. The binary interaction parameters *k_ij_* for R-32 resulted to be equal to 0.032 and 0.048 for PEBAX 1657 and PDMS, respectively.

### 3.3. Solubility of R-125

**Figure 2:**
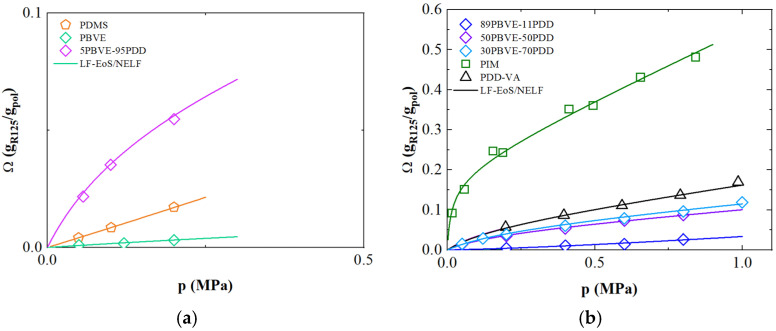
R-125 solubility isotherms as a function of pressure for different polymers (**a**) low pressure; (**b**) high pressure. The symbols represent the experimental data retrieved from literature [23,24,25,26,27,32], while continuous lines are obtained using LF/NELF model.

Figure 2 shows the solubility isotherms of R-125 (pentafluoroethane) as function of pressure of the same polymers inspected above, retrieved from [23,24,25,26,27,32]. The measurements were conducted at 303 K for PIM-1, while for the remaining materials, the temperature was set at 308 K.

As already observed for R-32, the solubility of R-125 increases by increasing the PDD content in the polymer matrix, since at low compositions of PDD, chain packing in the copolymer is more efficient due to a lower amount of bulky functional groups that significantly reduces the available free volume. Such condition is also reflected in the swelling coefficient *k_sw_* determined, which moves from 0.01 to 0.09 MPa^−1^ with PDD content within the copolymer (as reported in Table 2), highlighting the lower swellability of PBVE.

By applying the NELF approach on both the homopolymer and copolymers, a clear trend emerged: as the PDD content increased, the effective *k_ij_* decreased, even more than that of R-32 due to the fact that R-125 showed a sharper response to changes in copolymer composition.

Moreover, R-125 exhibits higher mass uptake than R-32 in fluorinated copolymers due to more favorable interactions given by the greater polarizability and higher fluorine-to-hydrogen ratio. The overall larger *k_sw_* obtained for R-125 with respect to R-32 may be attributed to the greater swelling of the polymer matrix induced by the larger penetrant molecules [23]. Relevantly, all these aspects are accurately described by the model approach considered, as the binary interaction parameter accounts for the repulsive/attractive interactions between penetrant molecules and polymer chains.

PIM-1 membranes presented higher sorption capacity compared to the other materials although the solubility of R-125 being just slightly higher than that of R-32. These results are in line with what expected based on penetrant condensability arguments often described by the gas critical temperature (see Table S2 in Supplementary Materials).

### 3.4. Solubility of R-134a

**Figure 3:**
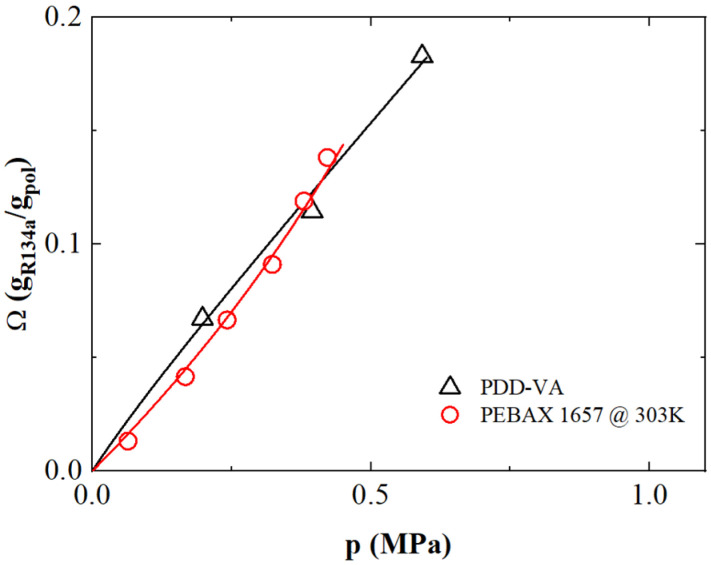
R-134a sorption isotherms as a function of pressure for co-polymers PDD-VA and PEBAX 1657. The symbols represent the experimental data retrieved from literature [25,26], while continuous lines are obtained using LF/NELF model.

The solubility isotherms of R-134a in PDD-VA copolymer and PEBAX 1657 are reported in Figure 3 as a function of pressure, as retrieved from the technical literature [25,26].

For this refrigerant gas, fewer experimental datasets are available in the literature, and only PDD-VA and PEBAX 1657 were inspected. Interestingly, although the two polymers are different in nature —being glassy the first one, and rubbery the second—the resulting solubility behavior is similar, displaying quite large gas uptake, reaching values up to 0.19 g_gas_/g_pol_ at 0.6 MPa for PDD-VA [25] and 0.15 g_gas_/g_pol_ at 0.4 MPa in PEBAX 1657 [32]. Remarkably, the thermodynamic model considered is able to describe accurately the sorption behavior for both copolymers as a function of pressure. For the PDD-VA copolymer, the model reproduces the experimental trend well as happens for the other refrigerant gases (despite its higher EoS coefficients), while the best fit for PEBAX 1657 is achieved by using a negative *k_ij_* parameter (Table 2).

### 3.5. Solubility of R-152a

**Figure 4:**
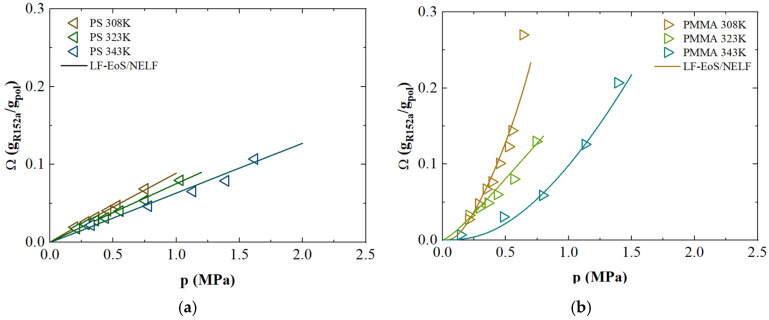
R-152a sorption isotherms as a function of pressure for (**a**) PS and (**b**) PMMA. The symbols represent the experimental data retrieved from literature [91], while continuous lines are obtained using LF/NELF model.

The analysis of R-152a (1,1 difluoroethane) solubility data in the literature revealed that only few polymers were inspected. In particular, Boudouris et al. [91] investigated the R-152a sorption in Polystyrene (PS) and Poly(methyl methacrylate) (PMMA) at various temperatures, namely 308, 323 and 343 K, as reported in Figure 4.

A significant gas uptake has been determined at all temperatures inspected for both materials, which decreases by increasing temperature, as usually observed in the case of gases and vapor sorption in polymers [92].

PS and PMMA are conventional glassy polymers, characterized by a moderate free volume that tends to increase during sorption, despite their non-equilibrium nature to accommodate the incoming gas molecules [91]. Based on their glass transition temperatures—373.6 K for PS and 378.15 K for PMMA—they should ideally be modeled using the Non-Equilibrium Lattice Fluid (NELF) framework. However, the high refrigerant uptake observed across all three experimental temperatures suggests the occurrence of a glass transition temperature (T_g_) shift during sorption, which may induce an earlier transition to the rubbery state. This behavior is particularly evident in PMMA (Figure 4b), which exhibits a convex sorption isotherm with increasing pressure—a trend attributed to solvent-induced plasticization. This plasticization leads to a progressive reduction in T_g_, making the polymer increasingly deviate from glassy behavior at higher pressures.

To verify this hypothesis, Chow’s model [93,94] was applied to both PS and PMMA (Figure S8 in Supplementary Materials). The results, reported in the Supplementary Materials, show that the T_g_ of PS decreases to 362 K upon sorption (ω = 0.097 g_R-152a_/g_pol_), while PMMA’s T_g_ drops even further to 319 K (at ω = 0.21 g_R-152a_/g_pol_), which is well below the testing temperatures thus supporting the occurrence of a plasticization-induced transition. By combining the Lattice Fluid model (applicable above the transition composition and pressure) with the Non-Equilibrium Lattice Fluid model (valid below that threshold), it was possible to accurately describe the sorption curves. This approach not only provided a good fit to the experimental data but also revealed simple, linear relationships for both *k_ij_* and *k_sw_* across the entire temperature range studied, as shown in Table 2. These results highlight the model’s effectiveness in capturing the system’s behavior under a broad set of operating conditions with minimal complexity.

### 3.6. Refrigerant Gases Transport in Polymers

Amorphous glassy polymers with tunable free volume are well-suited for optimizing gas transport in membranes. The combined use of the Standard Transport Model (STM) and Equation of State (EoS) has proven effective in describing the permeation behavior of various hydrofluorocarbons in selected polymers, enabling accurate prediction of permeability trends across a wide range of pressures and gas compositions.

**Figure 5:**
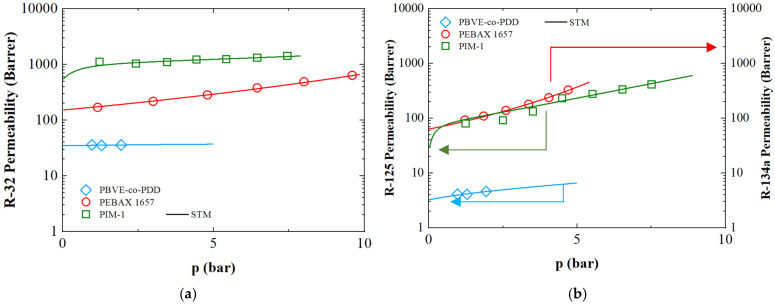
Experimental permeability data retrieved from literature for 30%PBVE-70%PDD, PIM-1 and PEBAX1657 as a function of pressure coupled with STM predictions (continuous lines). (**a**) Referred to permeability of R-32 gas, while (**b**) referred to R-125 (left *y*-axis) for 30%PBVE-70%PDD and PIM-1, and R-134a (right *y*-axis) for PEBAX 1657 [27,32,95].

Figure 5 reports the model analysis of the permeability of R32, R125, and R134a in PIM-1 and copolymers such as 30% PBVE-70% PDD and PEBAX 1657, using the experimental datasets retrieved from literature. The model accurately captures all different permeability behaviors, thanks to the determination of the mobility coefficient *L* (Equation (9)) and the plasticization factor *β*, as reported in (Table 3 for PIM and PBVE-PDD, and 4 for Pebax 1657).

Conversely to what happens for sorption (and for many hydrocarbons in which permeability increases with the number of carbon atoms), R-32 permeability appears to be greater than that of R-125 in the same material, which may be attributed to its smaller kinetic diameter and lower boiling point (Table S2), resulting in lower condensability whose effect is more than offset by the faster diffusion.

Regarding the plasticization factor *β*, the highest value is observed for R-125 in PIM-1, followed by the fluorinated polymers, suggesting that this gas induces greater swelling, as was observed also for *k_sw_*. The highest *L_∞_* for R-32 is observed for PEBAX 1657, likely due to its rubbery, flexible phase, which facilitates faster penetrant transport. In contrast, PIM-1, presents the lowest *L_∞_* for both R-32 and R-125, highlighting the influence of molecular size and free volume on mobility coefficients as permeation appears to be a diffusion-driven process.

Permeation behavior in 30% PBVE-70% PDD polymer is higher for R-32 and a sharp contrast in ***L_∞_*** between R-32 and R-125, pointing to selective diffusivity behavior that can be conveniently leveraged for separation.

To gain a deeper understanding of the transport behavior of the fluorinated refrigerant gases through the polymers, a comparison is performed between the permeability of the fluorinated gases and their nonfluorinated hydrocarbon analogs, such as methane and ethane, respectively. Compared to fluorinated refrigerants, the analogous hydrocarbons generally exhibited lower permeability and weaker interactions with polymer matrices. This behavior can be attributed to their lower condensability and lack of dipolar interactions. For instance, different grades of PEBAX present higher permeability coefficients of R-32 and R-134a with respect to methane and ethane due to their higher electric dipolar moment and critical temperature that leads to greater polymer-penetrant interactions. Similar consideration can be made also for PIM-1, PBVE-co-PDD and PDMS in which methane permeability resulted to lay, respectively, between 400 [96] to 860 [97] Barrer, from 1 to 30 [31] and from 800 [20] to 1000 [98] Barrer, while that of R-32 exceeds 1200 [32] for PIM-1, 300 for PBVE-co-PDD (according to the PDD percentage in the matrix) [95], and 2700 Barrer for PDMS [23].

In fact, while R-32 (but more in general refrigerant gases) shows strong affinity and high solubility due to its polarizability and fluorine content, methane presents a lower solubility, which is not counterbalanced by the smaller kinetic diameter thus resulting in lower permeability values.

### 3.7. Refrigerant Gases Sorption and Transport in Supported Ionic Liquid Membranes: A Case Study

Membrane performance for refrigerant gas separation can be improved by incorporating an Ionic Liquid into the polymer matrix, enabling selective interactions that facilitate gas transport. It is reported by Palomar et al. [99] that the absorption process in ILs is kinetically controlled so that using low-viscosity ILs would enhance gas diffusion coefficient.

Thermodynamic and transport models can indeed be applied to Ionic Liquid and SILM allowing the prediction of the interaction between the HFC gas and the polymer + immobilized selective ILs. Although more advanced equations of state, such as iPC-SAFT [100,101], explicitly account for specific interactions like hydrogen bonding and ionic character, the Lattice Fluid model has been selected in this work due to its relative simplicity and its demonstrated adequacy in capturing the essential thermodynamic behavior of Ionic Liquid, when no other ionic species are involved.

Specifically, a case study from the literature was selected where both solubility and permeability data are available, over a broad range of pressure, for refrigerant gases in supported Ionic Liquid membranes (SILMs), namely Pebax1657/[C_2_mim][BF_4_] and Pebax1657/[C_2_mim][SCN]. In this case, the two Ionic Liquids were treated as plasticizer compound added to the material matrix (meaning no chemical interaction with the polymeric chains) and, by applying the mixing rules, the characteristic parameters (p*, T*, and ρ*) were readily calculated for the blends, enabling a more comprehensive evaluation of the interaction between refrigerant gases, the polymer, and the immobilized selective Ionic Liquids.

**Figure 6:**
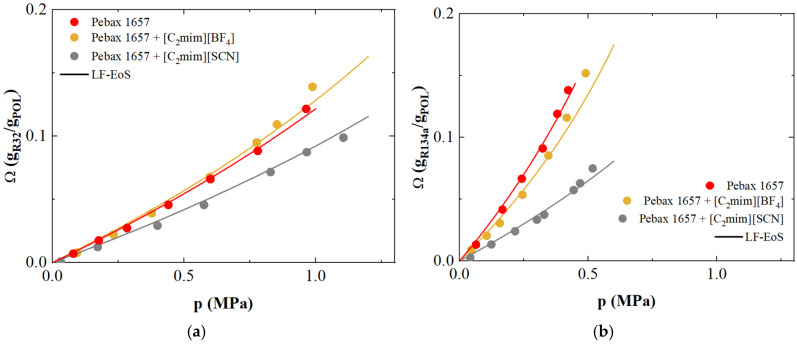
Comparison between experimental solubility data and Sanchez-Lacombe Lattice Fluid EoS phase resolution of (**a**) R-32 in Pebax 1657 + 40 wt.% [C_2_mim][BF_4_] and Pebax 1657 + 40 wt.% [C_2_mim][SCN], and (**b**) (**a**) R-134a in Pebax 1657 + 40 wt.% [C_2_mim][BF_4_] and Pebax 1657 + 40 wt.% [C_2_mim][SCN]. Symbols are experimental data retrieved from literature [102], lines represent LF EoS.

Comparisons of solubility data with calculated values are illustrated in Figure 6, showing that the method is quite accurate: as one can see, the solubility curves prove that the present LF-EoS is applicable for methyl–imidazolium based Ionic Liquid systems for properties description and solvent design.

As already observed by Pardo et al. [26,102], the presence of Ionic Liquids in PEBAX does not significantly improve membrane performance for refrigerant gases. The immobilization of 40 wt.% [C_2_mim][SCN] within the polymer matrix causes a decrease in the sorption capacity of all gases with respect to that of the pure Pebax 1657, suggesting that the larger molar volume of SCN promotes a blockage phenomenon, as clearly visible in Supplementary Materials from Figures S9–S13 in which are compared the solubility isotherms in ILs [BF_4_] and [SCN] for different refrigerant gases. This behavior is also evident from the analysis of the energy interaction parameters for the penetrant + SILM systems, which shows the lowest *k_ij_* value for pure Pebax 1657 with R-134a (Table 4). In contrast, the highest *k_ij_* is observed for Pebax containing 40 wt.% [C2mim][SCN], which also exhibits the lowest gas uptake, indicating a repulsive interaction between the components.

**Figure 7:**
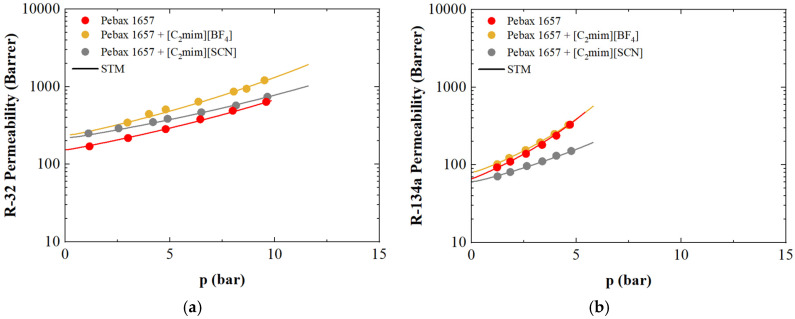
Model description of (**a**) R-32 in Pebax 1657 + 40 wt.% [C_2_mim][BF_4_] and Pebax 1657 + 40 wt.% [C_2_mim][SCN], and (**b**) R-134a in Pebax 1657 + 40 wt.% [C_2_mim][BF_4_] and Pebax 1657 + 40 wt.% [C_2_mim][SCN]: symbols are experimental data retrieved from literature [102], lines represent STM.

On the other hand, the enhancement of R-32 transport in the presence of Ionic Liquids (ILs) is clearly evident, as reflected by the increased mobility coefficients compared to the neat polymer matrix. In contrast, for the larger R-134a molecule, permeability through the Pebax membrane containing 40 wt.% [C2mim][SCN] decreases relative to neat Pebax 1657 (Figure 7).

These observations indicate that HFC gas permeability through IL-containing membranes is favored by strong enthalpic interactions with fluorinated ILs, while smaller molar volume ILs with lower anion fluorination degrees tend to diminish permeability.

Notably, the modeling approach captures this behavior effectively: Pebax + 40 wt.% [C2mim][SCN] exhibits the highest plasticization factor and infinite dilution diffusion coefficients (*L_∞_*) for both gases (4.0 × 10^−7^ cm^2^/s for R-32 and 1.5 × 10^−7^ cm^2^/s for R-134a). This suggests that gas molecules, once absorbed, tend to plasticize the polymer matrix, thereby enhancing their diffusion mobility.

Conversely, in the absence of Ionic Liquids, pure Pebax 1657 shows significantly lower gas mobility coefficients (2.3 × 10^−7^ cm^2^/s for R-32 and 7.5 × 10^−8^ cm^2^/s for R-134a), indicative of a more constrained diffusion environment within the neat polymer matrix.

## 4. Conclusions

The present study investigated the application of Equation of State (EoS) and Standard Transport Model (STM) modeling approach to the analysis of solubility and permeability behavior of hydrofluorocarbon gases—namely R-32, R-125, R-134a, and R-152a—in a wide range of amorphous polymer matrices, including homopolymers (such as PIM-1, PMMA, PDMS and PS) and copolymers (PDD-VA, Pebax and PBVE-PDD). The proposed approach has been successfully applied to several gas-polymers systems and, also in case under examination, has confirmed its ability to describe experimental behavior of refrigerant gases based on few adjustable parameters, providing a valid interpratation of the structure–property relationships that governs the gas–polymer interactions.

To apply this methodology, the following steps are required:

- Retrieve the main pure components parameters of the models considered (SL EoS or NELF) through the use of a series of experimental data, such as pVT or VLE data, of the pure components, as well as for, the glassy polymers, their density and glass transition temperature and, in case of copolymers, information on chemical composition.
- Fit the Sanchez–Lacombe equation of state or its non-equilibrium extension to a single sorption isotherm to extract polymer-specific parameters and interaction coefficients (*k_ij_* and *k_sw_*).
- Combine these thermodynamic results with the Standard Transport Model (STM)—that incorporates thermodynamic solubility and concentration-dependent mobility coefficients—to estimate permeability, which can be validated against experimental transport data.
- Use the obtained modeling tools to describe and predict gas solubility, diffusivity and permeability in a wide range of experimental conditions, possibly defining general guidelines to describe the expected behavior of the gas-polymer system of interest.

In the present case, the sketched approach revealed that fluorinated refrigerants exhibit markedly different solubility profiles depending on both polymer chemistry and gas structure, but still consistent with usual trends related to condensability and molecular size. This is the case of R-134a and R-125 generally showing higher uptake than its smaller and more volatile cousin, R-32.

The modeling framework effectively captured variations in gas sorption behavior arising from polymer composition and microstructure. For example, as clearly visible for both R-32 and R-125 in PBVE–PDD copolymers, increasing PDD content correlates with a reduced polymer–gas interaction energy parameter and a higher swelling coefficient, indicating that copolymer composition can be strategically tuned to modulate both permeability and swelling. The observed variations in *k_ij_* and *k_sw_* with PDD content highlight their utility as design parameters for polymer–refrigerant systems.

It was also demonstrated that the SL EoS/NELF framework, together with STM, resulted to be suitable even for more complex systems such as SILM, where the Ionic Liquid phase governs the sorption of R-32 and R-125 in the matrix.

Compared to conventional light hydrocarbons, fluorinated gases typically exhibit higher permeability when the membrane possesses sufficient fractional free volume and compatibility, such as in perfluorinated copolymers (e.g., PBVE-co-PDD).

Overall, this integrated modeling approach provides a systematic tool for evaluating and designing high-performance polymeric membranes for the separation and recycling of low-GWP refrigerants. These results support the strategic development of tailored materials for sustainable membrane-based gas separations in the refrigerant industry.

## Figures and Tables

**Table 1:** LF Characteristic parameters for polymers and refrigerant gases investigated in this work.

Materials	T* [K]	p* [MPa]	ρ* [g/cm^3^]	Reference
PBVE	650	315	2.190	[28]
89PVBE-11PDD	649	309	2.177	This work
50PBVE-50PDD	646	286	2.132	This work
30PBVE-70PDD	644	274	2.108	This work
5PBVE-95PDD	642	258	2.077	This work
75PDD-25VA	579	287	1.854	This work
PDD	632	274	2.068	This work
VA	459	415	0.955	[88]
PIM-1	872	523	1.438	[85]
PS	750	360	1.090	[68]
PMMA	695	560	1.270	[53]
PDMS	560	355	1.200	[61]
PEBAX 1657	664	684	1.200	This work, pVT data from [61]
C_2_mim [BF_4_]	650	580	1.385	This work, pVT data from [89]
C_2_mim [SCN]	700	540	1.175	This work, pVT data from [90]
R32	339	633	1.46	This work, VLE data from [60]
R125	324	342	1.91	This work, VLE data from [60]
R134a	349	460	1.78	This work, VLE data from [60]
R152a	360	495	1.27	This work, VLE data from [60]

**Table 2:** Binary parameters calculated from solubility analysis of the LF/NELF model (binary interaction parameter k_ij_ and swelling coefficient k_sw_) for the polymer of interest in this work. The majority of the data are reported for sorption isotherms at 308 K, whilst * refers to sorption isotherms fit at 303 K. Finally, PS and PMMA present a linear behavior with temperature.

	R-32		R-125		R-134a		R-152a	
	*k_ij_*	*k_sw_* [MPa^−1^]	*k_ij_*	*k_sw_* [MPa^−1^]	*k_ij_*	*k_sw_* [MPa^−1^]	*k_ij_*	*k_sw_* [MPa^−1^]
PBVE	0.100	0.010	0.160	0.01	-	-	-	-
89PVBE-11PDD	0.095	0.015	0.150	0.05	-	-	-	-
50PBVE-50PDD	0.080	0.030	0.070	0.08	-	-	-	-
30PBVE-70PDD	0.075	0.030	0.060	0.08	-	-	-	-
5PBVE-95PDD	0.049	0.050	0.054	0.09	-	-	-	-
75PDD-25VA	0.057	0.058	0.051	0.110	0.071	0.250	-	-
PIM-1 *	0.06	0.09	0.080	0.20	-	-	-	-
PS	-	-	-	-	-	-	−7.8 × 10^−4^ T + 0.29	−9.9 × 10^−5^ T + 0.12
PMMA	-	-	-	-	-	-	−5.7 × 10^−4^ T + 0.21	−2.5 × 10^−3^ T + 1.03
PDMS	0.040	(0.020) ^#^	0.055	(0.070) ^#^	-	-	-	-
PEBAX1657 *	0.048	(0.120) ^#^	-	-	−0.002	(0.200) ^#^	-	-

^#^ Swelling coefficients in brackets for PDMS and PEBAX 1657 have been predicted considering the NELF approach, although the polymers are in rubbery state.

**Table 3:** STM parameters used for the analysis of gas transport.

Materials	30%PBVE-70%PDD		PIM-1	
	R-32	R-125	R-32	R-125
***L_∞_* [cm^2^/s]**	7.7 × 10^−8^	2.5 × 10^−9^	3.0 × 10^−8^	3.5 × 10^−10^
***β***	21	24	19	25.5

**Table 4:** LF EoS and STM parameters used for the description of solubility and permeability isotherms of Pebax + ILs.

Materials	Pebax 1657		Pebax + 40 wt.% [C_2_mim][BF_4_]		Pebax + 40 wt.% [C_2_mim][SCN]	
	R-32	R-134a	R-32	R-134a	R-32	R-134a
***k_ij_***	0.048	−0.002	0.001	0.008	0.02	0.038
***L_∞_* [cm^2^/s]**	2.3 × 10^−7^	7.5 × 10^−8^	3.0 × 10^−7^	1.0 × 10^−7^	4.0 × 10^−7^	1.5 × 10^−7^
***β***	23	20	25	22	26	28

## Data Availability

Data are available on request.

## Supplementary Materials

The following supporting information can be downloaded at https://www.mdpi.com/article/10.3390/polym17162169/s1; Table S1. LF EoS parameters; Table S2. Physical and chemical properties of refrigerant gases [1]; Figure S1. Best-fit of the Vapor-Liquid equilibrium for the determination of LF EoS characteristic parameters for (a) R-32, (b) R-125, (c) R.134-a and (d) R.152a taken from [1]; Figure S2. Best-fit of pVT data for the determination of LF EoS characteristic parameters for [C_2_mim][BF4] taken from [2]; Figure S3. Best-fit of pVT data for the determination of LF EoS characteristic parameters for [C_2_mim][SCN], taken from [3]; Figure S4. High pressure prediction in R-32; Figure S5. High pressure prediction in R-125; Figure S6. High pressure prediction in R-134a; Figure S7. High pressure prediction in R-152a; Figure S8. Chow’s model applied to R-152a in (a) PS and (b) PMMA. Experimental parameters for Chow’s model are taken from literature for the two polymers [7,8], respectively; Figure S9. R32-solubility in [C_2_mim][BF4]]. Symbols represent experimental data at different temperatures taken from [9], while lines correspond to LF-EoS; Figure S10. R-125 solubility in [C_2_mim][BF4]. Symbols represent experimental data at different temperatures taken from [9], while lines correspond to LF-EoS; Figure S11. R-134a solubility in [C_2_mim][BF4]. Symbols represent experimental data at different temperatures taken from [9], while lines correspond to LF-EoS.; Figure S12. R-32 solubility in [C_2_mim][SCN]. Symbols represent experimental data at different temperatures taken from [10], while lines correspond to LF-EoS; Figure S13. R-134a solubility in [C_2_mim][SCN]. Symbols represent experimental data at different temperatures taken from [10], while lines correspond to LF-EoS.

## Abbreviations

The following abbreviations are used in this manuscript:

R-32	Bifluoromethane
R-125	Pentafluoroethane
R-134a	1,1,1,2-Tetrafluoroethane
R-152a	1-1 Difluoroethane
CH_4_	Methane
C_2_H_6_	Ethane
PDMS	Polydimethylsiloxane
PIM-1	Polymer of Intrinsic Microporosity
PEBAX	poly(ether-block-amide)
PBVE	perfluoro(butenyl vinyl ether)
PDD	perfluoro(2,2-dimethyl-1,3-dioxole)
VA	Vinyl Acetate
SILM	Supported liquid membrane
SL-EoS	Sanchez-Lacombe Equation of State
NELF	Non-Equilibrium Lattice Fluid
STM	Standard Transport Model
