# Supplementary material for: Description of Gas Transport in Polymers: Integrated Thermodynamic and Transport Modeling of Refrigerant Gases in Polymeric Membranes
Source: Polymers (Basel). 2025 Aug 8;17(16):2169. doi: 10.3390/polym17162169 (PMC12389465; doi:10.3390/polym17162169)
Supplement: Supplementary file 1 [file polymers-17-02169-s001.zip › polymers-3773007-supplementary.pdf]

Supporting Information

Prediction of Gas Transport in Polymers: Thermodynamic and Kinetic Insights into the Behavior of Refrigerant Gases

Matteo Minelli, Marco Giacinti Baschetti and Virginia Signorini *

Department of Civil, Chemical, Environmental and Materials Engineering, Alma Mater Studiorum, University of Bologna, via Terracini 28, 40131 Bologna, Italy; matteo.minelli@unibo.it (M.M.); marco.giacinti@unibo.it (M.G.B.)
* Correspondence: virginia.signorini2@unibo.it

Table S1: LF EoS parameters

**Pure component *i***		
M_i	Molar mass of component *i*	
ρ_i	Density of component *i*	
v_i^*	Molar volume of a lattice cell of component *i*	$v_i^* = \frac{RT_I^*}{p_i^*}$
r_i^0	Number of lattice cells occupied by a molecule of pure component *i*	$r_i^0 = \frac{M_i}{\rho_i^* v_i^*}$
ω_i	Mass fraction of i	
φ_i	Volume fraction of component *i* at close packed conditions	$\varphi_i = \frac{\omega_i/\rho_i^*}{\sum_i^N \omega_i/\rho_i^*}$
ε_i	Non-bonded interaction energy between two lattice cells occupied by component *i*	
L	Mobility coefficient	
L_0	Infinite dilution mobility coefficient	
β	Plasticization factor	
α	Thermodynamic factor	
$\tilde{T}_i$	Reduced temperature of component *i*	$\tilde{T}_i = \frac{T}{T_i^*}$
$\tilde{p}_i$	Reduced pressure of component *i*	$\tilde{p}_i = \frac{p}{p_i^*}$
$\tilde{\rho}_i$	Reduced density of component *i*	$\tilde{\rho}_i = \frac{\rho_i}{\rho_i^*}$
T^*	Characteristic temperature	$T^* = \frac{\varepsilon^*}{k_b}$
p^*	Characteristic pressure	$p^* = \frac{\varepsilon^*}{v^*}$
ρ^*	Closed-packed density	$\rho^* = \frac{M}{rv^*}$
**Multicomponent mixtures**		
k_{ij}	Binary interaction parameter between components *i* and *j*	
ρ^*	Characteristic density of the mixture	$\frac{1}{\rho^*} = \sum_i^N \frac{\omega_i}{\rho_i^*}$

p^*	Characteristic pressure of the mixture	$p^* = \sum_i^N \varphi_i p_i^* - \sum_i^{N-1} \sum_{j>i}^N \varphi_i \varphi_j \Delta p_{ij}^*$
Δp_{ij}^*	Binary parameter	$\Delta p_{ij}^* = p_i^* + p_j^* - 2(1 - k_{ij})\sqrt{p_i^* p_j^*}$
T^*	Characteristic temperature of the mixture	$T^* = \frac{p^*}{r} \sum_i^N x_i r_i^0 \frac{T_i^*}{p_i^*}$
v^*	Average close-packed molar volume in the mixture	$v^* = \frac{T^* R}{p^*}$
r_i	Number of lattice cells occupied by a molecule in the mixture	$r_i = \frac{r_i^0 v_i^*}{v^*}$
$\tilde{T}$	Reduced temperature of the mixture	$\tilde{T} = \frac{T}{T^*}$
$\tilde{p}$	Reduced pressure of the mixture	$\tilde{p} = \frac{p}{p^*}$
$\tilde{\rho}$	Reduced density of the mixture	$\tilde{\rho} = \frac{\rho}{\rho^*}$

Figure S1 reports the best-fit of the experimental data for the Vapor-Liquid equilibrium for the different refrigerant gases retrieved from Perry's Chemical Engineering Handbook [1], used to calculate the characteristic parameters T*, p* and ϱ* required to solve the phase equilibrium in the Sanchez Lacombe Lattice Fluid EoS.

Similarly, Figures S2 and S3 report the experimental standard Pressure-volume-temperature data and the model fit used to determine the T*, p* and ϱ* for Ionic Liquids [C_2mim][BF_4] [2] and [C_2mim][SCN] [3].

On the other hand, the characteristic parameters for the polymers investigated in this work are retrieved from literature, while that of copolymers were obtained from literature data of the pure component coupled with the appropriate mixing rules [4–6].

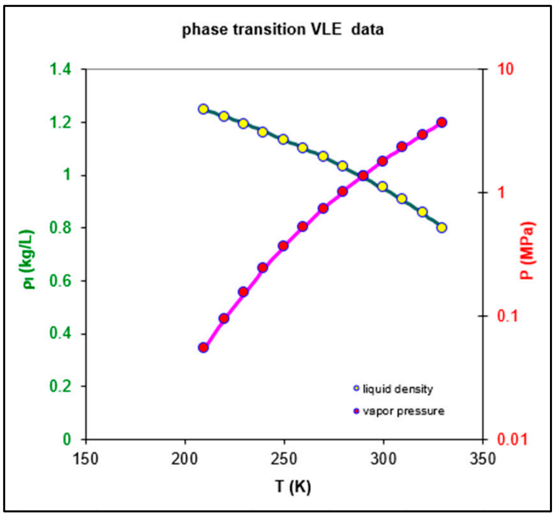

R-32

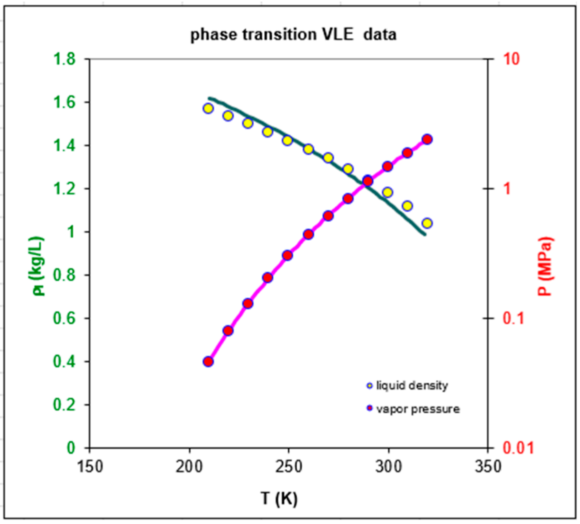

R-125

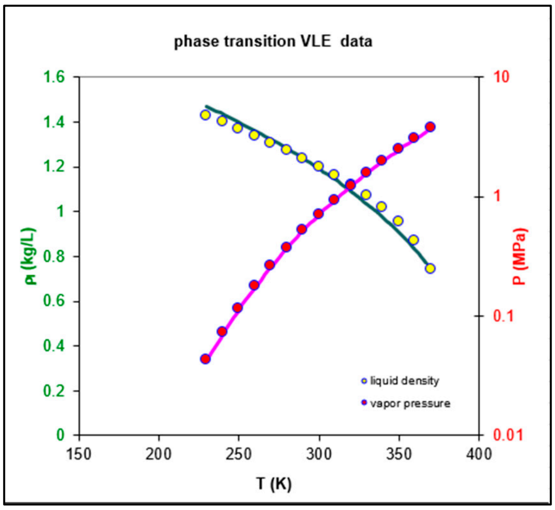

R-134a

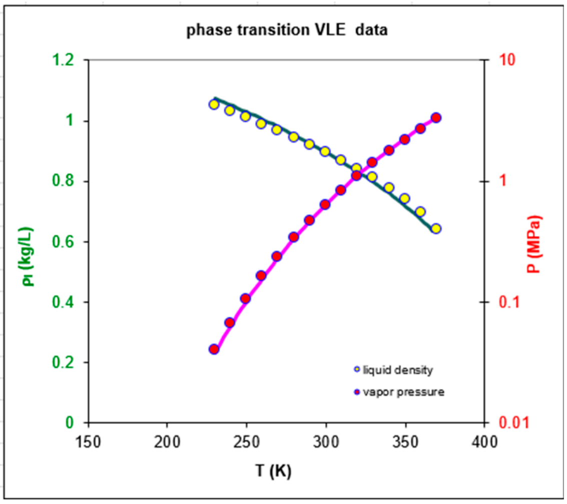

R-152a

Figure S1. Best-fit of the Vapor-Liquid equilibrium for the determination of LF EoS characteristic parameters for (a) R-32, /b) R-125, (c) R.134-a and (d) R.152a taken from [1]

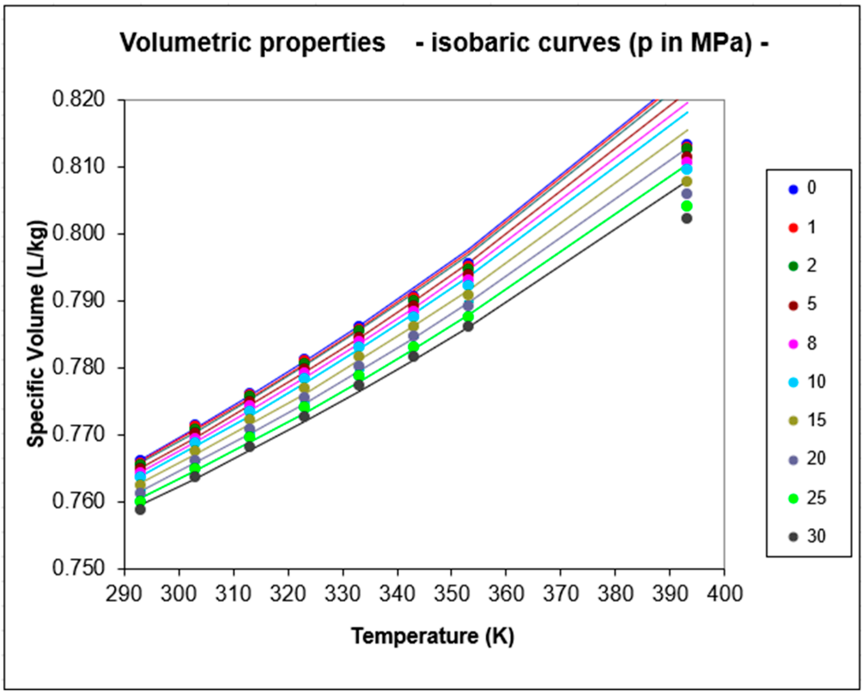

Figure S2. Best-fit of pVT data for the determination of LF EoS characteristic parameters for [C_2mim][BF_4] taken from [2]

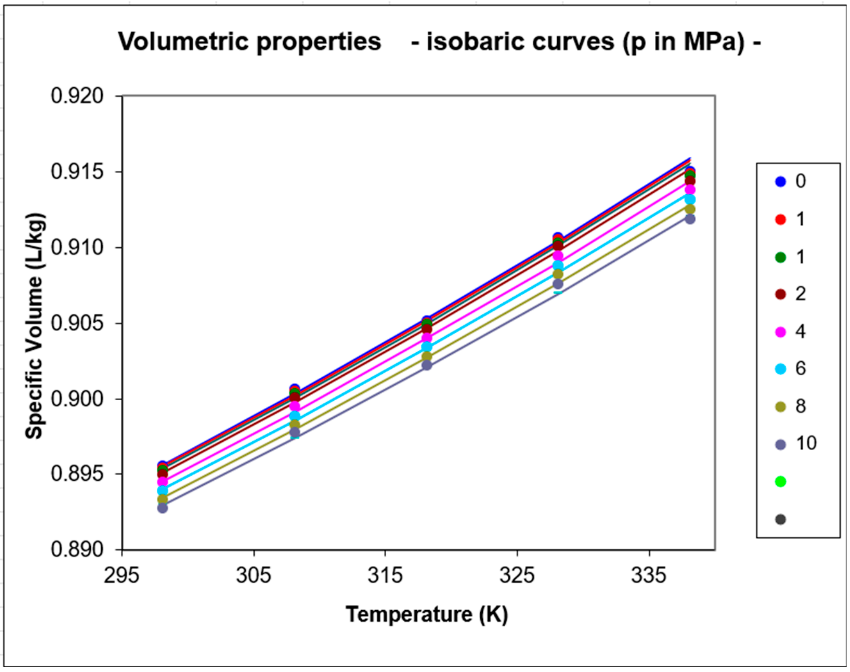

Figure S3. Best-fit of pVT data for the determination of LF EoS characteristic parameters for [C_2mim][SCN], taken from [3]

Table S2: Physical and chemical properties of refrigerant gases [1]

Compound	R-32	R-125	R-134a	R-152a
Name	Difluoromethane	1,1,1,2,2-pentafluoroethane	1,1,1,2,-Tetrafluoroethane	1,1-difluoroethane
Molecular formula	CH_2F_2	CHF_2CF_3	$C_2H_2F_4$	CHF_2CH_3
Molecular Mass [g/mol]	52.02	120.03	102.03	66.06
Normal boiling point [°C]	-51.65	-48.5°C	-26.05	-25.15
p_c [MPa]	5.78	3.63	4.06	4.52
T_c [°C]	78.11	66.18	101.5	112.85

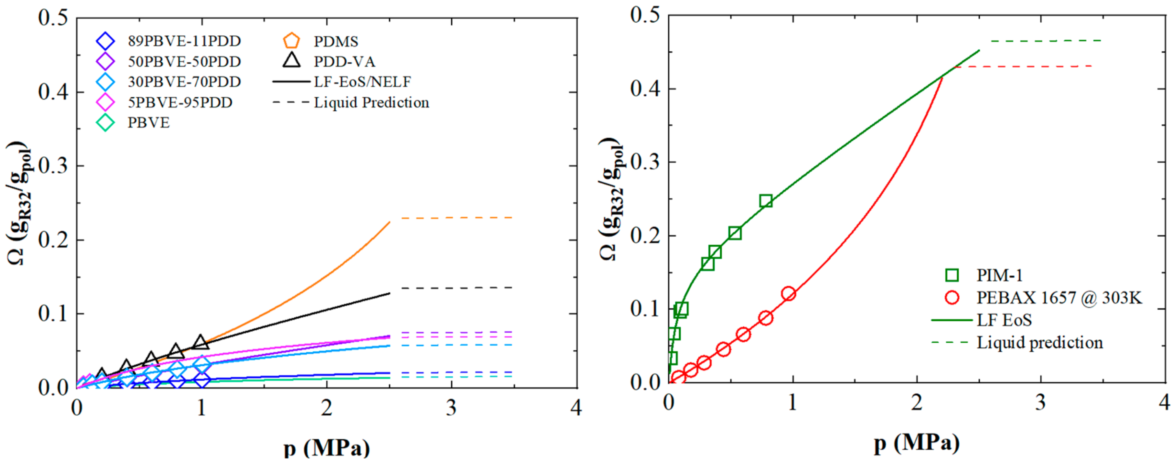

Figure S4. High pressure prediction in R-32

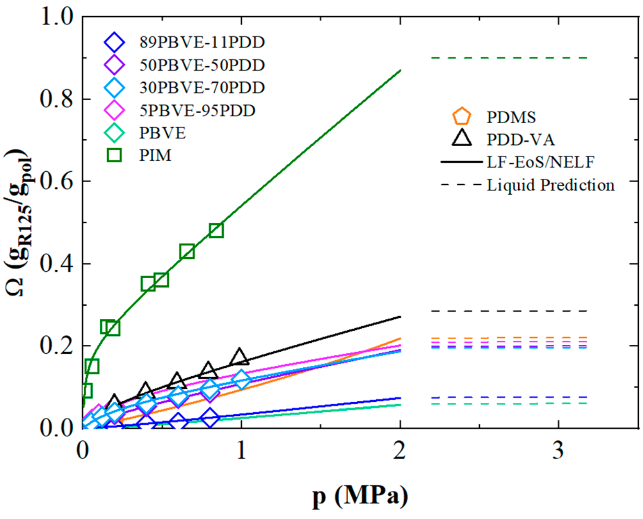

Figure S5. High pressure prediction in R-125

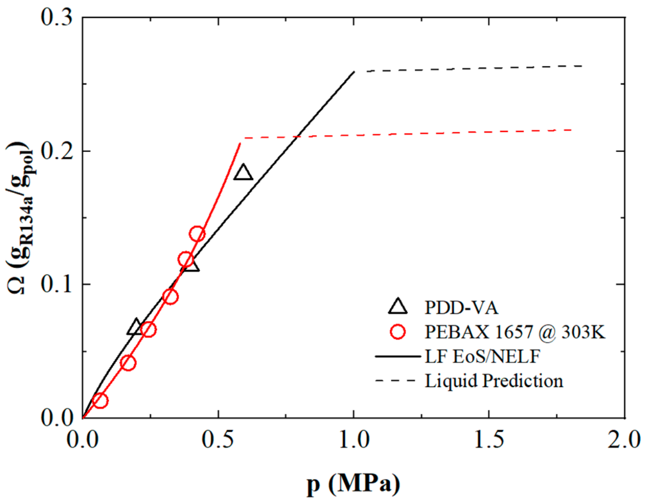

Figure S6. High pressure prediction in R-134a

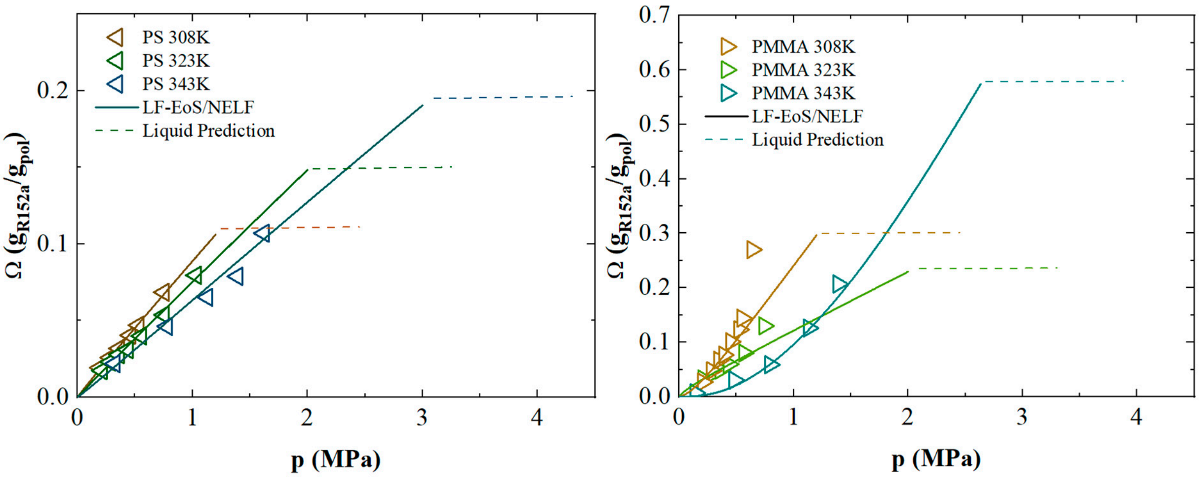

Figure S7. High pressure prediction in R-152a

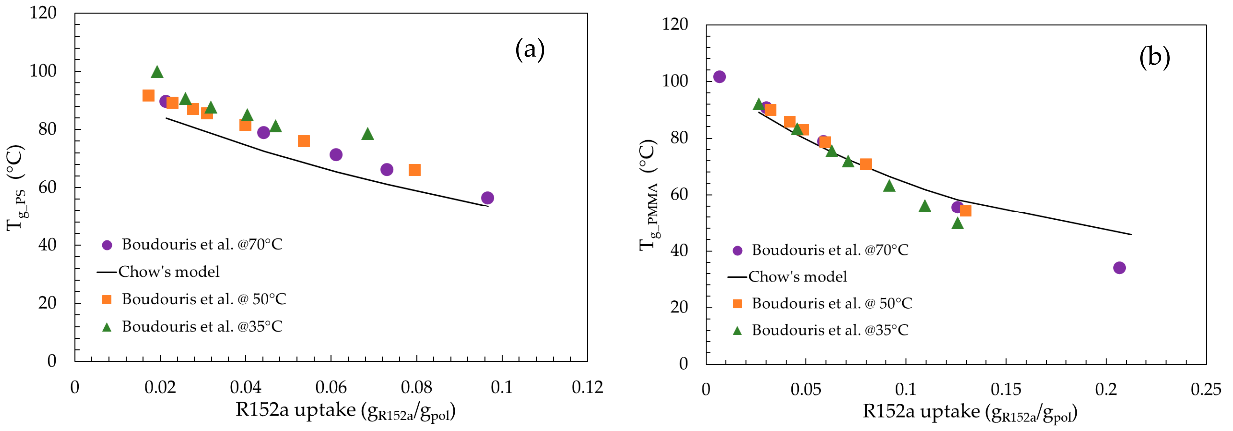

Figure S8. Chow's model applied to R-152a in (a) PS and (b) PMMA. Experimental parameters for Chow's model are taken from literature for the two polymers [7,8], respectively.

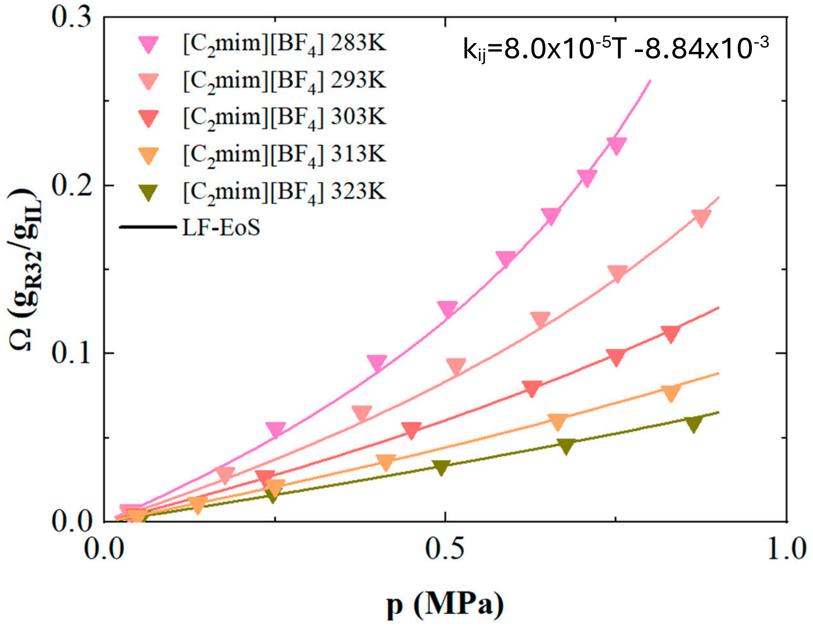

Figure S9. R32-solubility in [C2mim][BF4]]. Symbols represent experimental data at different temperatures taken from [9], while lines correspond to LF-EoS.

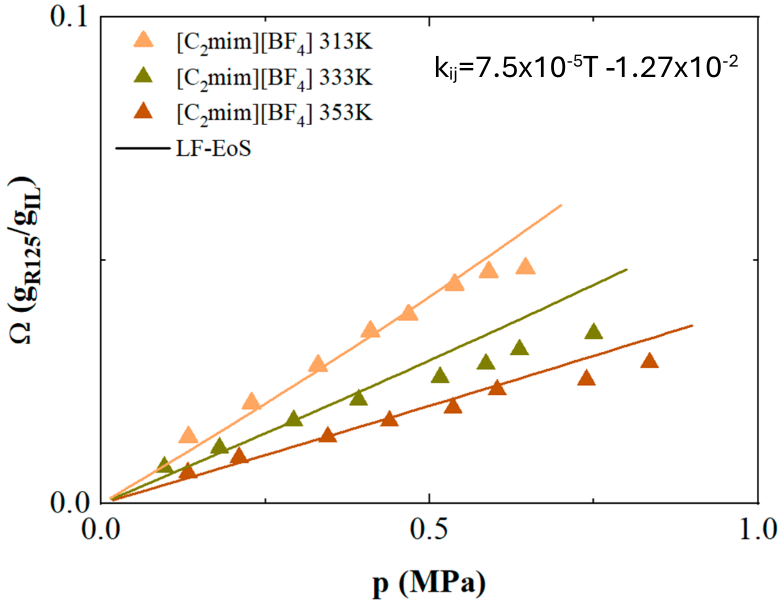

Figure S10. R-125 solubility in [C2mim][BF4]. Symbols represent experimental data at different temperatures taken from [9], while lines correspond to LF-EoS.

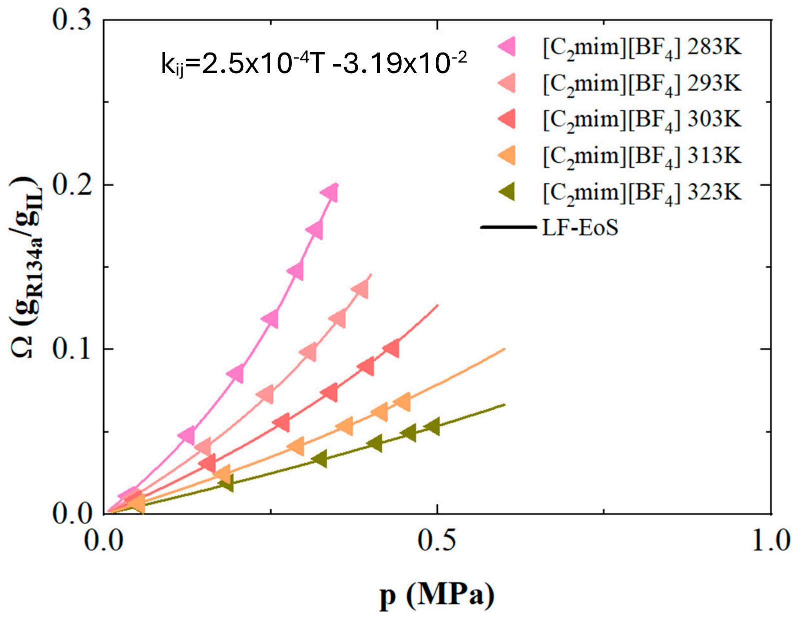

Figure S11. R-134a solubility in [C2mim][BF4]. Symbols represent experimental data at different temperatures taken from [9], while lines correspond to LF-EoS.

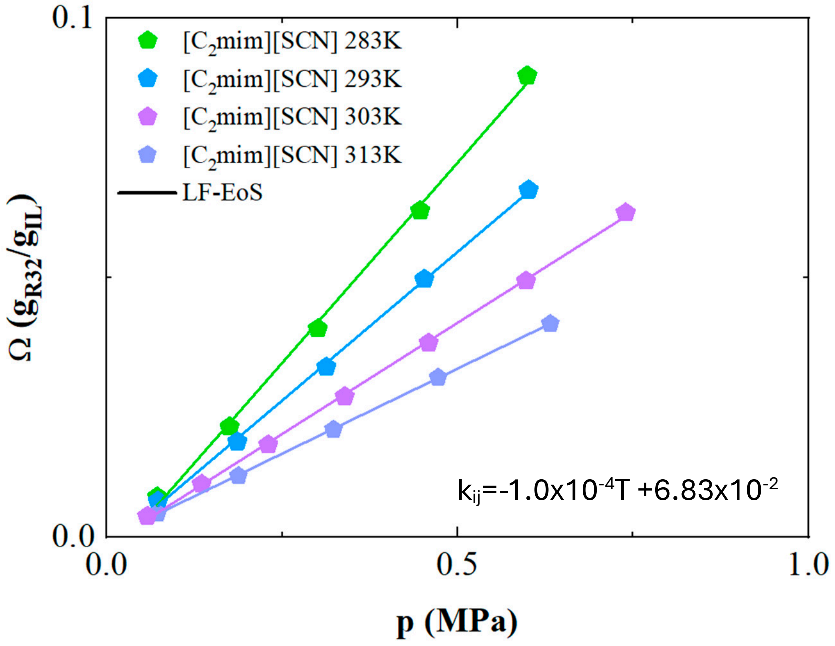

Figure S12. R-32 solubility in [C2mim][SCN]. Symbols represent experimental data at different temperatures taken from [10], while lines correspond to LF-EoS

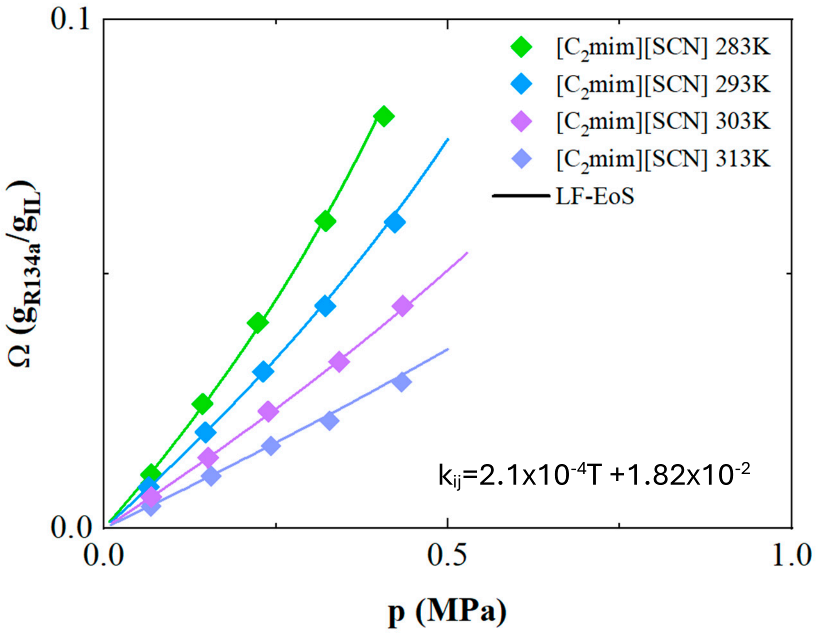

Figure S13. R-134a solubility in [C_2mim][SCN]. Symbols represent experimental data at different temperatures taken from [10], while lines correspond to LF-EoS

Reference

1. Green, Dr.D.W.; Southard, Dr.M.Z. *Perry's Chemical Engineers' Handbook*; McGraw-Hill Education, 2019; ISBN 9780071834087.

2. Gardas, R.L.; Freire, M.G.; Carvalho, P.J.; Marrucho, I.M.; Fonseca, I.M.A.; Ferreira, A.G.M.; Coutinho, J.A.P. PρT Measurements of Imidazolium-Based Ionic Liquids. *J Chem Eng Data* **2007**, *52*, 1881–1888, doi:10.1021/JE700205N/SUPPL_FILE/JE700205N-FILE002.PDF.

3. Królikowska, M.; Hofman, T. Densities, Isobaric Expansivities and Isothermal Compressibilities of the Thiocyanate-Based Ionic Liquids at Temperatures (298.15–338.15 K) and Pressures up to 10 MPa. *Thermochim Acta* **2012**, *530*, 1–6, doi:10.1016/J.TCA.2011.11.009.

4. Sanchez, I.C. Relationships between Polymer Interaction Parameters. *Polymer (Guildf)* **1989**, *30*, 471–475, doi:10.1016/0032-3861(89)90016-5.

5. Lacombe, R.H.; Sanchez, I.C. Statistical Thermodynamics of Fluid Mixtures. *Journal of Physical Chemistry* **1976**, *80*, 2568–2580, doi:10.1021/j100564a009.

6. Eitouni, H.B.; Balsara, N.P. Thermodynamics of Polymer Blends. *Physical Properties of Polymers Handbook* **2007**, 339–356, doi:10.1007/978-0-387-69002-5_19.

7. Marti, E.; Kaisersberger, E.; Moukhina, E. Heat Capacity Functions of Polystyrene in Glassy and in Liquid Amorphous State and Glass Transition: DSC and TMDSC Study. *J Therm Anal Calorim* **2006**, *85*, 505–525, doi:10.1007/S10973-006-7745-5.

8. Soldera, A.; Metatla, N.; Beaudoin, A.; Said, S.; Grohens, Y. Heat Capacities of Both PMMA Stereomers: Comparison between Atomistic Simulation and Experimental Data. *Polymer (Guildf)* **2010**, *51*, 2106–2111, doi:10.1016/J.POLYMER.2010.03.003.

9. Asensio-Delgado, S.; Pardo, F.; Zarca, G.; Urtiaga, A. Vapor-Liquid Equilibria and Diffusion Coefficients of Difluoromethane, 1,1,1,2-Tetrafluoroethane, and 2,3,3,3-Tetrafluoropropene in Low-Viscosity Ionic Liquids. *J Chem Eng Data* **2020**, *65*, 4242–4251, doi:10.1021/ACS.JCED.0C00224/ASSET/IMAGES/LARGE/JE0C00224_0008.JPEG.

10. Asensio-Delgado, S.; Pardo, F.; Zarca, G.; Urtiaga, A. Enhanced Absorption Separation of Hydrofluorocarbon/Hydrofluoroolefin Refrigerant Blends Using Ionic Liquids. *Sep Purif Technol* **2020**, *249*, 117136, doi:10.1016/J.SEPPUR.2020.117136.
